# Significance of E-cadherin and Vimentin as epithelial-mesenchymal transition markers in colorectal carcinoma prognosis

**DOI:** 10.17179/excli2020-1946

**Published:** 2020-06-26

**Authors:** Zohreh Niknami, Ahad Muhammadnejad, Alireza Ebrahimi, Zahra Harsani, Reza Shirkoohi

**Affiliations:** 1 Department of Genetics, Faculty of Science, Islamic Azad University, Damghan, Iran; 2 Cancer Biology Research Center, Cancer Institute of Iran, Tehran University of Medical Sciences, Tehran, Iran; 3 Department of Hematology, Faculty of Medical Sciences, Tarbiat Modares University, Tehran, Iran; 4 Department of Cell and Molecular Biology, Islamic Azad Medical University of Tehran, Tehran, Iran

**Keywords:** colorectal cancer, E-cadherin, Vimentin, immunohistochemistry

## Abstract

Colorectal cancer is the most common malignancy of the gastrointestinal tract with very high mortality. One of the most distinguishing features for the establishment of an epithelial-mesenchymal transition phenotype is the alteration of mesenchymal markers and structural adhesion proteins. We investigated the level of Vimentin and E-cadherin expression in relation to invasion and metastasis on colorectal cancer patients. Tissue specimens were collected consecutively from thirty-nine colorectal carcinoma patients during surgeries. The patients were diagnosed and treated between 2013 and 2016. In order to histological staging, tissue sections were prepared from formalin-fixed paraffin-embedded blocks and stained with Hematoxylin and Eosin. Also for evaluating the epithelial-mesenchymal transition markers, E-cadherin and Vimentin, all patient samples were stained and detected via immunohistochemistry, and afterwards the results were analyzed to determine whether these markers could be useful prognostic markers for predicting colorectal cancer patient outcomes. The expression of Vimentin as a mesenchymal marker along with rising grade of cancer, pathological stages, and metastasis to regional lymph nodes increased furthermore, in cancers with vascular invasion, Vimentin value was high. Reversely, the expression of E-cadherin with climbing grade, stages and colon cancer categories decreased and also in cancers with vascular invasion reduced. Variation of the markers had no relation to age and sex. In summary, along with cancer progression level of Vimentin expression varies inversely with E-cadherin expression and by increasing metastasis and invasion the Vimentin expression elevates. Further evaluation in this area might lead to a good method for predicting progressive clone cancer.

## Introduction

It is well demonstrated that colorectal cancer (CRC) is one of the highly aggressive malignant tumors. CRC is third cancer following lung and prostate cancers in men and second cancer in women after breast cancer in the world (Armaghany et al., 2012[1]). It is also a cancer with a very high mortality, as its mortality rate is the third highest among all cancers (Ye et al., 2015[23]) and the course of the disease results in death in about one of two CRC patients. Over 1.2 million new CRC cases, approximately 800,000 deaths are recorded annually (Jemal et al., 2011[7]; Sun et al., 2016[18]). The study of CRC syndromes has greatly assisted understanding of the molecular pathogenesis underlying sporadic CRC. Lynch syndrome (3-5 % of colorectal cancer), an autosomal dominant inherited disorder, is a disease in which pathogenesis is influenced by environmental exposures, genetic and epigenetic alterations (Sinicrope et al., 2016[17]). Some risk factors include lifestyle, dietary factors, side-effects of medical interventions, older age, family history and inherited genetic disorders (Lin, 2009[9]). Tumor development risk has increased through one of the most complex cellular events as a multi-step cascading process involving infinite proliferation, invasion, and immigration (Huang et al., 2016[6]). The prognosis of CRC is related to the stage of cancer at diagnosis time, as with a five years survival rate of 90 % at early diagnosis and less than 10% when metastasis (Yiu and Yiu, 2016[24]). About 20 % of CRC patients have tumor metastasis at diagnosis time and so lost the best treatment opportunity. Surgery, chemotherapy, radiotherapy and biological target therapy as treatment methods have increased life quality of patients, however, 50 % of patients pass away after 2 years of treatment by recurrence or metastasis (Ye et al., 2015[23]). Recent investigations have focused on the epithelial-mesenchymal transition (EMT) in cancer invasion and metastasis (Toiyama et al., 2013[20]). At the first time, EMT has been defined by Elizabeth Hay in 1968, as a biological process in which epithelial cells can down-regulate epithelial characteristics and acquire mesenchymal characteristics (Xiao et al., 2015[22]). Also, it is a key step during embryonic development and epithelial tumor metastasis that changes cell-cell and cell-extracellular matrix interactions by altering cellular molecular levels, causing transmigration cells, and then leading to metastasis (Zhao et al., 2014[25]). Overall, in the EMT process the expression and function of epithelial markers such as E-cadherin decline and mesenchymal markers like Vimentin increase (Ye et al., 2015[23]). These researches on the molecular genomic targets and biomarkers underpinning EMT regulation hold tremendous potential in identifying the subset of patients that are at highest risk of developing metastasis in CRC.

Several factors and genes are associated with the process of tumor angiogenesis, invasion, growth, and metastasis in CRC. One of the most distinguishing features for the establishment of an EMT phenotype is up-regulated expression of mesenchymal markers and down-regulated expression of structural adhesion proteins (Micalizzi and Ford, 2009[11]). According to the studies, it is clear that Vimentin is a multifunctional protein and its ability to interact with a large number of proteins makes it a potential regulator of several different physiological functions (Satelli and Li, 2011[16]). This protein is responsible to maintain cell shape, the integrity of the cytoplasm, stabilizing cytoskeletal interactions and involving an immune response (Ogrodnik et al., 2014[13]). It operates as an organizer of a number of critical proteins involved in attachment, migration, and cell signaling. Vimentin expresses during embryonic development and predominantly in the primitive streak stage, also it is present in connective tissue mesenchymal cells, CNS, muscles, fibroblasts, endothelial cells, macrophages, neutrophils, and lymphocytes (Otsuki et al., 2011[14]; Satelli and Li, 2011[16]). 

E-cadherin is a cell-cell junction protein and a classical marker of epithelial cell (Xiao et al., 2015[22]) that plays an important role in epithelial cells adhesion and the tissue architecture maintenance. E-cadherin is a member of Cadherins superfamily (Transmembrane glycoproteins), that is in epithelial cells moreover other subclasses are neural (N-cadherin), placental (P-cadherin) and vascular endothelial cadherin (VE-cadherin). (Tsanou et al., 2008[21]). Therefore, this study was established due to our assumption that Vimentin and E-cadherin biomarkers play roles in the CRC pathological process. To determine the level of the EMT markers in CRC tissue compared to together, the levels of Vimentin and E-cadherin were measured. Consequently, we hope to find a new diagnostic and treatment approach of colorectal cancer.

## Materials and Methods

### Patients 

This study was performed as a cross-sectional study for 39 CRC patients during four years from 2013 to 2016. Thirty-nine fresh samples (N=39) were collected consecutively from Tumor bank of Iran Cancer Institute of Iran, Imam Khomeini Hospital Complex (Tehran, Iran). Informed written consent was obtained from patients and all procedures were performed according to the guidelines of scientific research ethical committee of Tehran University of Medical Science. The tissues were analyzed via immunohistochemistry. Tissue slides were reviewed by a pathologist and were scored underlying modified Allerd scoring system (Table 1(Tab. 1)). Also, staging was determined according to last version tumor, lymph nodes, metastasis (TNM) classification for colon cancer of the American Joint Committee on Cancer (AJCC) and TNM Classification of Malignant Tumours, eighth edition of Union for International Cancer Control (UICC). None of the patients in this study had received preoperative chemotherapy or radiotherapy.

### Immunohistochemistry 

Tissue specimens were divided to separate parts for pathological assays. In order to histological staging, tissue sections were prepared from formalin-fixed paraffin-embedded (FFPE) blocks and stained with Hematoxylin and Eosin (*H&E*). Finally, tissues were classified by a pathologist.

For immunohistochemistry, we used Master Polymer Plus detection system (Master Diagnostica, Granada, Spain). The paraffin sections were first dewaxed in xylene and rehydrated in decreasing alcohol solutions and then hydrated with distilled water. Endogenous peroxidase activity was blocked by applying 100 µl peroxidase blocking reagent to each sample and incubated for 10 min at room temperature (RT) in darkness. This was followed by three washes with Tris-buffered saline (TBS) for 5 min. 100 µl of the primary antibody amplifier master were used for each sample and the samples were incubated in RT for 15 min and then washed three times with TBS for 5 min. In the next step 100 µl of the master polymer plus HRP were used for each sample and the samples were incubated in RT for 30 min and then rinsed as in the previous step.

**Figure 1:**
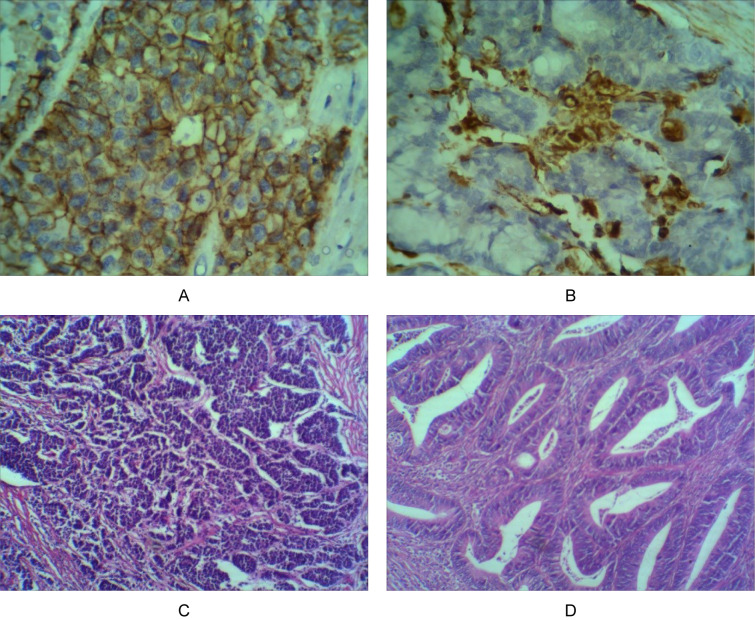
Several photographs of pathological examination. (A) E-cadherin expression 400X, (B) Vimentin expression 400X, (C) High grade CRC 100X, (D) Low grade CRC 100X.

In the visualization step "Immunostain", after preparation of the chromogenase solution as manufacturer's order, the samples were covered with the solution and incubated for 5 min in RT and then washed freely with distilled water three times for 5 min. Afterwards, DAB enhancer was used for 2 min at RT and washed by distilled water 3 times and finally, the samples were covered with Hematoxylin stain for a minute and rinsed a lot by distilled water and were dehydrated and mounted. There are four pictures which are applied for pathological examination in Figure 1(Fig. 1).

### Statistical analysis

The statistical procedures were included Mean ratio (M), Standard Deviation (SD), Confidence Intervals (95 % CI), Standard Error of Mean (SEM). Mean values and associations between discrete variables were assessed using the ANOVA and the Chi-square tests. The significance level was set at *P* value <0.05. All mathematical analyses were performed using the statistical package for the social sciences software (SPSS Inc. v. 21).

## Results

### Demographic data analysis

In our study, there were 17 male patients, 22 female patients, with the age ranging from 27 to 79 years old; the mean age was about 57 years. All CRC patients had no chemotherapy and were in grade I (n=9; 23.1 %), grade II (n=23; 59 %), grade III (n=7; 17.9 %). The primary site of samples were included cecum (n=6), rectum (n=11), Sigmoid Colon (n=6), Right side (n=9), Left side (n=1) and NOS (n=6) (see Table 2(Tab. 2)).

### Comparison Vimentin and E-cadherin relative to other clinical parameters 

**Figure 2:**
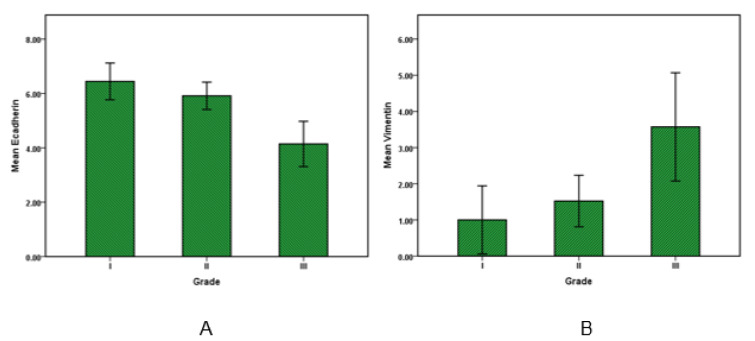
E-cadherin and Vimentin expression in colorectal cancer. Bar graphs illustrate the values of E-cadherin (A) and Vimentin (B) in low to high-grade carcinoma. Results are expressed as the mean ± SD.

For evaluating the correlation of Vimentin and E-cadherin expression with sex, age, the primary site of cancer, tumor stage, grade of cancer, pT category (primary tumor) and pN Category (regional lymph nodes) and also vascular invasion were used for statistical tests. Afterwards, the analysis showed that amounts of Vimentin and E-cadherin expression have no statistically significant relationship to sex, age and primary location of clone cancer. E-cadherin levels in grade I and II were higher than grade III (*P* value <0.05), this shows the amount of E-cadherin expression as an epithelial marker decline by cancer progression. Moreover, the Vimentin expression along with grade number increased and its amount in grade III was significantly higher than grade I and II (*P* value <0.05). Overall, in accordance with growth of grade, Vimentin expression increased and E-cadherin expression decreased as shown in Figure 2(Fig. 2).

**Figure 3:**
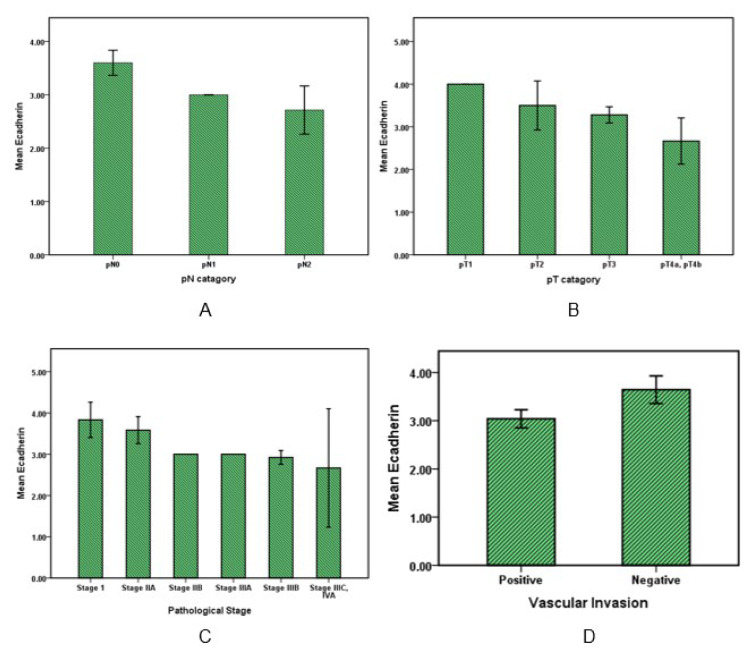
E-cadherin expression in colorectal cancer. Bar graphs illustrate the values of E-cadherin with pN and pT pathological categories (A, B) and also with pathological stages of cancer (C). The graph D shows E-cadherin variation in cancers with or without vascular invasion.

Data analysis (Figure 3A, 3B(Fig. 3)) showed that E-cadherin expression in evaluating with pN category (regional lymph nodes) lowered according to increasing metastasis in the number of regional lymph nodes so that E-cadherin expression in pN0 was higher than pN1 and pN2 (*P* value <0.05). And also in relation to the pT category with increase in invasion into other organs or structures, the E-cadherin rate was significantly reduced (P value <0.05).

E-cadherin expression level with increasing pathological stages of the disease (Figure 3C(Fig. 3)) decreased (*P* value <0.05). Moreover, E-cadherin in patients with vascular invasion was significantly (*P* value <0.05) lower than patients that had no sign of vascular invasion (Figure 3D(Fig. 3)). 

**Figure 4:**
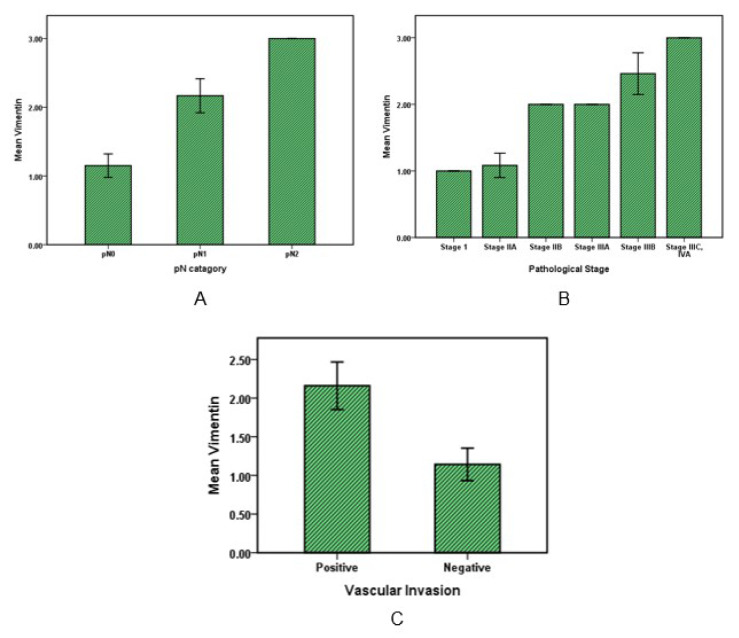
Vimentin expression in colorectal cancer. Bar graphs illustrate the values of Vimentin with pN pathological category (A) and with pathological stages of cancer (B). Graph C shows Vimentin variations in cancers with or without vascular invasion.

Statistical analysis on Vimentin values with pN category and stages of the cancer patients showed that Vimentin has significantly (*P* value˂0.001) increased in cancer specimens with metastasis in 1 to 3 regional lymph nodes (pN1) relative to specimens with no regional lymph node metastasis (pN0) and also average of Vimentin in cancers with four or more metastasis regional lymph nodes was significantly (*P* value˂0.001) higher than all (Figure 4A(Fig. 4)). Furthermore, in evaluation with pT category of cancer by increasing tumor invasion from submucosa (pT1) to tumor invasion to other organs or structure like visceral peritoneum, the Vimentin value did not increase significantly (*P* value>0.05).

Vimentin expression was low in the first stage of cancer, while a significant increase was observed in the other stages up to stage four (*P* value˂0.05). Moreover, according to Figure 4C(Fig. 4), the Vimentin amount in cancers with vascular invasion was about 2-fold higher (mean 2.2±0.75) than other cancers without Vascular invasion (mean=1.14± 0.36) (*P* value˂0.01).

## Discussion

Colorectal cancer is the most common malignancy of the gastrointestinal tract that begins with the transformation of normal epithelial cells to an adenoma, proceeding to *in situ* carcinoma, and eventually to an invasive and metastatic tumor. Although, it is one of the well-studied cancers in recent years, the therapeutic process is not yet perfect. In a morphogenetic process in which epithelial cells miss their characteristics and gain mesenchymal properties during the progression of cancer. The EMT process is characterized by loss of epithelial markers such as E-cadherin and gain of mesenchymal markers such as Vimentin (Gout and Huot, 2008[5]). It was thought that the key role of CRC is involved in genetic alterations and molecular expressions. Further genetic and epigenetic studies were conducted on the CRC (Theiss et al., 2014[19]). Growing evidence suggests that some biomarkers play an important role in CRC development, progression and metastasis (Brenner and Rennert, 2005[2]; Niknami et al., 2017[12]; Ou et al., 2013[15]; Satelli and Li, 2011[16]; Toiyama et al., 2013[20]). Vimentin and E-cadherin are responsible for maintaining cell shape, cytoplasm integrity, stabilizing cytoskeletal interactions, cell signaling, migration and cell adhesion (Micalizzi and Ford, 2009[11]; Ogrodnik et al., 2014[13]). These markers were evaluated in various epithelial cancers including prostate cancer, gastrointestinal tumors, CNS tumors, breast cancer, malignant melanoma, lung cancer and other cancers (Satelli and Li, 2011[16]). Many studies and researches have reported that for most cancers, as the cancer progresses, Vimentin has increased (Niknami et al., 2017[12]; Xiao et al., 2015[22]), while E-cadherin has decreased (Bruun et al., 2014[3]; Kolijn et al., 2015[8]; Xiao et al., 2015[22]; Ye et al., 2015[23]).

Essentially, expression of Vimentin is mainly associated with the metastatic phenotype and poor prognosis of the disease outcome. In this study, the Vimentin amount of tumors was associated with a growth of grade and stage of cancer, as well as with cancer progression to metastasis stage Vimentin increased. This data is in accordance with our past study on CRC that the mRNA level of Vimentin gene increased in low tumor grade (I-III) (Niknami et al., 2017[12]) and also it is according to other studies like Liu and colleagues (2017[10]).

Data analysis also showed that Vimentin has increased with the growth of regional lymph nodes (pN category) in cancer tissues but in primary tumor (pT category) vimentin rate was not significant. While vimentin overexpression in cancer correlates with a high invasion of tumor growth (Satelli and Li, 2011[16]), our results showed that with more metastasis vimentin increases. Furthermore, along with vascular invasion Vimentin has highly increased, whereas, parts of metastasis process include proliferation of a primary tumor, local invasion of detached cells, intravasation in a capillary, and tumor cell survival in blood circulation (Gout and Huot, 2008[5]); in this study the increase of Vimentin is according to metastasis process. Chen and colleagues showed Vimentin expressed cancerous colon tissue (Chen et al., 2005[4]). 

The most crucial steps in the tumor cell metastasis are destroying cell-cell junctions, degrading the cell matrix, and activating pathways that control the cytoskeletal dynamics of cancer cells (Gout and Huot, 2008[5]). E-cadherin is an important extracellular matrix (ECM) glycoprotein that plays an important role in cell adhesion, migration, cancer growth and development (Tsanou et al., 2008[21]). As before, most studies reported that E-cadherin was reduced in advanced CRC (Bruun et al., 2014[3]; Tsanou et al., 2008[21]). In this study, data analysis revealed that E-cadherin has decreased with the growth of primary tumor steps (pT category) and regional lymph nodes (pN category) in cancer tissues. Moreover, with grade and stage progression, and vascular invasion, E-cadherin decreased.

And in final, Vimentin and E-cadherin have no significant correlation with age, gender, and tumor location.

Overall, according to our study Vimentin as a mesenchymal marker accelerates and E-cadherin as a structural adhesion protein reduces in colorectal cancer. The simultaneous analysis of these two proteins can help determine the progression of the cancer. However, more research is needed to investigate the function of these proteins in the EMT process and their metastasis in colorectal carcinoma for therapeutic purposes.

## Notes

Alireza Ebrahimi and Reza Shirkoohi (Cancer Biology Research Center, Cancer Institute of Iran, Tehran University of Medical Sciences, Tehran, Iran, Zip Code: 1419733141; Tel: 0098-21-66914545, Fax: 0098-21-66581638, E-mail: rshirkoohi@tums.ac.ir) equally contributed as corresponding authors.

## Conflict of interest

The authors declare that they have no conflict of interest.

## Figures and Tables

**Table 1:**
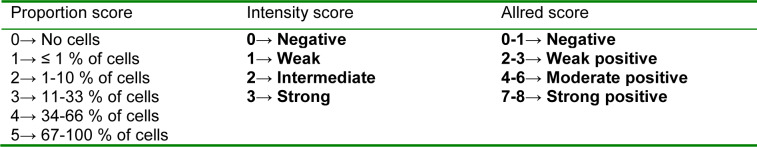
Modified Allred scoring system

**Table 2:**
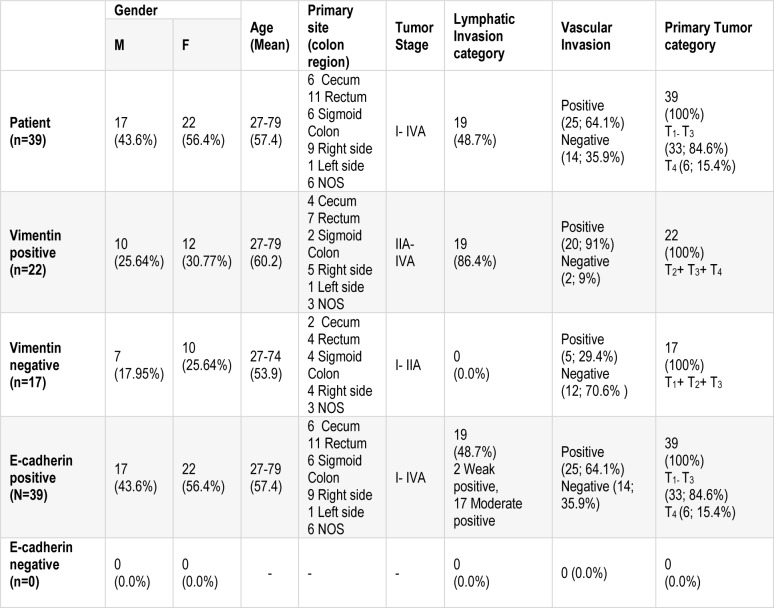
A demographic table including patient's age, gender, tumor size, and tumor stage
